# Kv1 channels and neural processing in vestibular calyx afferents

**DOI:** 10.3389/fnsys.2015.00085

**Published:** 2015-06-02

**Authors:** Frances L. Meredith, Matthew E. Kirk, Katherine J. Rennie

**Affiliations:** ^1^Department of Otolaryngology, University of Colorado School of MedicineAurora, Colorado, USA; ^2^Department of Physiology and Biophysics, University of Colorado School of MedicineAurora, Colorado, USA

**Keywords:** potassium conductance, gravity, gerbil, utricle, crista, dendrotoxin, margatoxin

## Abstract

Potassium-selective ion channels are important for accurate transmission of signals from auditory and vestibular sensory end organs to their targets in the central nervous system. During different gravity conditions, astronauts experience altered input signals from the peripheral vestibular system resulting in sensorimotor dysfunction. Adaptation to altered sensory input occurs, but it is not explicitly known whether this involves synaptic modifications within the vestibular epithelia. Future investigations of such potential plasticity require a better understanding of the electrophysiological mechanisms underlying the known heterogeneity of afferent discharge under normal conditions. This study advances this understanding by examining the role of the Kv1 potassium channel family in mediating action potentials in specialized vestibular afferent calyx endings in the gerbil crista and utricle. Pharmacological agents selective for different sub-types of Kv1 channels were tested on membrane responses in whole cell recordings in the crista. Kv1 channels sensitive to α-dendrotoxin and dendrotoxin-K were found to prevail in the central regions, whereas K^+^ channels sensitive to margatoxin, which blocks Kv1.3 and 1.6 channels, were more prominent in peripheral regions. Margatoxin-sensitive currents showed voltage-dependent inactivation. Dendrotoxin-sensitive currents showed no inactivation and dampened excitability in calyces in central neuroepithelial regions. The differential distribution of Kv1 potassium channels in vestibular afferents supports their importance in accurately relaying gravitational and head movement signals through specialized lines to the central nervous system. Pharmacological modulation of specific groups of K^+^ channels could help alleviate vestibular dysfunction on earth and in space.

## Introduction

During spaceflight astronauts experience altered gravity conditions and resulting sensorimotor dysfunction. The impairment of balance, movement, coordination and spatial orientation has been coined Space Adaptation Syndrome (SAS). SAS results in significant disorientation and motion sickness, but the underlying physiological mechanisms are unclear (Rizzo-Sierra and Leon-Sarmiento, 2011). Animal studies have shown that an intact vestibular system is required for the induction of motion sickness (Yates et al., 1998) and it is hypothesized that altered patterns of activity in vestibular afferent fibers during gravity transitions may drive sensory conflicts resulting in SAS symptoms. A better understanding of how the peripheral vestibular system functions during normal and altered gravity conditions should lead to identification of more selective pharmacological targets to alleviate vestibular dysfunction and motion sickness (Soto and Vega, 2010; Lackner, 2014).

K^+^ constitutes an ion of major importance in the inner ear. K^+^-rich endolymph bathes the apical surfaces of hair cells, whereas basal surfaces are bathed in perilymph. Potassium ions enter hair cells through mechanically-sensitive channels in stereocilia and exit hair cells via ion channels in the basolateral membrane. The importance of K^+^ channels in the inner ear is emphasized by channel mutations which have profound consequences resulting in deafness and vestibular disorders (Zdebik et al., 2009). Clearly K^+^ channels are involved in fine-tuning the electrical activity of hair cells and afferent fibers necessary for the normal sensory perception of vestibular signals. In order to target specific groups of ion channels in vestibular hair cells and their afferent fibers, a better understanding of their biophysical properties and distribution is required. Modulating specific K^+^ channels by enhancing or decreasing their activity could help alleviate symptoms arising from unwanted vestibular signals and ease the transitions of astronauts through changing gravitational environments.

To elucidate the roles of ion channels in the processing of signals in the vestibular periphery we have developed slice preparations of rodent crista and utricle which allow electrophysiological comparisons between central zones (CZ) and peripheral zones (PZ). Here we focus on specialized vestibular primary afferent terminals that form calyx endings (calyces) on type I hair cells. We show that K^+^ currents in calyces from PZ of the crista and extrastriolar regions of the utricle demonstrate more inactivation than calyces in CZ and striolar regions. Underlying zonal differences in K^+^ channel populations were investigated. Afferent spike timing differs between zones suggesting different mechanisms for encoding vestibular stimuli exist (Goldberg, 2000; Eatock and Songer, 2011). However, similarities between otolith organs and cristae suggest mechanisms driving firing characteristics are conserved between vestibular end organs. Previous work has identified several different conductances in vestibular calyx terminals, including those mediated by KCNQ K^+^ channels, inactivating A-type K^+^ channels, hyperpolarization-activated cyclic nucleotide-sensitive (HCN) channels and small conductance calcium-activated K^+^ channels (Hurley et al., 2006; Rennie and Streeter, 2006; Dhawan et al., 2010; Meredith et al., 2011, 2012; Horwitz et al., 2014). In this study, we probe the electrophysiological expression of Kv1 channels in calyx endings. The Kv1 channel subfamily has several members constituting Kv1.1 through Kv1.7 and Kv1 channels can co-assemble with other members of the same family. In vestibular calyx terminals we find that a dendrotoxin-sensitive conductance prevails in central epithelial regions, suggesting contributions from Kv1.1 and/or Kv1.2 channels. Margatoxin-sensitive currents are more prevalent in peripheral regions and may be mediated by Kv1.3 or Kv1.6 channels. Mutations in Kv1 channels are known to result in ataxic disorders (Jen, 2008; Jan and Jan, 2012) and their presence in vestibular primary afferents suggests they are also important for the accurate relay of gravitational and head movement signals to the central nervous system.

## Materials and Methods

### Tissue Preparation

Mongolian gerbils (*Meriones unguiculatus*) of both sexes and aged between postnatal days (P) 17–29 were used. Intraperitoneal injections of ketamine (70 mg/kg) and xylazine (3 mg/kg) were used to induce anesthesia. Following decapitation the vestibular sensory organs (cristae and utricles) were removed. Procedures adhered to protocols approved by the University of Colorado’s Institutional Animal Care and Use Committee.

Techniques for obtaining calyx recordings were similar to those described previously in crista (Meredith and Rennie, 2015), with the addition of recordings from calyces in utricular slices. Cristae were trimmed, embedded in a solution of 4% low gelling temperature agarose (2-Hydroxyethylagarose, Type VII, Sigma-Aldrich, St. Louis, MO, USA) and sliced transversely using a Vibratome 3000 EP^TM^ (Saint Louis, MO, USA) as described previously (Meredith and Rennie, 2015). Otoliths were gently removed with a stainless steel minutien pin prior to embedding and slicing utricles in 4% agarose gel. Vestibular slices ranged in thickness from 100–120 μM. Slices were secured with a small weight, bathed in Leibovitz’s L-15 medium (pH 7.4–7.45, osmolality 300–305 mmol/kg) and viewed under an Olympus upright microscope (BX50WI or BX51WI) with water immersion objectives (×40 or ×60) and differential interference contrast (DIC) optics. The crista epithelium has previously been divided into concentric areas, with the central third of the sensory epithelium area designated as the central zone (CZ) and surrounding areas defined as the PZ (Lindeman, 1969; Desai et al., 2005a; Lysakowski and Goldberg, 2008). Cup-shaped afferent calyx terminals were seen surrounding the basal regions of type I hair cells in both zones. After establishing a gigaohm seal with a patch electrode on a calyx, images of the slice were recorded with a digital camera (Rolera, QImaging) and QCapture Pro 6.0 software for subsequent confirmation of zonal location.

All utricle and all crista CZ recordings were made from slices. In a few cases, instead of slicing, micro-dissection scissors were used to cut the peripheral ends of the crista and the severed peripheral end sections (“cut ends”) were held down with a minutien pin for recordings (*n* = 10 calyces). Recordings were also made from dissociated PZ calyces (*n* = 6) in some experiments (Rennie and Streeter, 2006). Here, sensory end organs were incubated in a high Mg^2+^/low Ca^2+^ solution containing (in mM): NaCl (135), KCl (5), MgCl_2_ (10), CaCl_2_ (0.02), HEPES (10), and D-glucose (3), pH 7.4 with NaOH and osmolality 300–305 mmol/kg, at 37°C for 10–30 min following their removal from the ear. After washing in L-15/BSA, cells were mechanically dissociated by drawing a fine probe across the separated peripheral crista end.

### Electrophysiological Recordings

A micropipette puller (Sutter Instruments, San Rafael, CA, USA) was used to make patch electrodes whose tips were heat polished on a Narishige MF 830 microforge (Narishige International USA, East Meadow, NY, USA). Silicone elastomer (Sylgard 184, Dow Corning, Midland, MI, USA) was applied close to the electrode tip. Electrode solution contained (in mM): KF (115), KCl (10), NaCl (2), HEPES (10), D-glucose (3), MgCl_2_ (2), and EGTA (10), pH 7.4 with KOH (21–28 mM), osmolality 300–305 mmol/kg (adjusted with sucrose or mannitol). Electrode open tip resistance was 2–7 MΩ. A patch amplifier (Axopatch-1D or Axopatch 200B, Molecular Devices, Sunnyvale, CA, USA), connected to a PC through an AD converter (Digidata 1320A or 1440A, Molecular Devices, Sunnyvale, CA), was used to obtain whole cell recordings at room temperature (21–24°C). Clampex software pClamp (v8 or 10) was used for data acquisition and analysis. Data were low-pass filtered online at 2 or 5 kHz and the sampling rate ranged from 2–10 kHz. Liquid junction potentials were corrected during data analysis. 4-aminopyridine (4-AP), margatoxin and dendrotoxins were obtained from Sigma-Aldrich (St. Louis, MO, USA). 10, 10-bis(pyridin-4-ylmethyl)anthracen-9-one (XE991) and CP-339, 818 were obtained from Tocris Bioscience (Ellisville, MO, USA). In most experiments, tissue was perfused continuously with L-15 and drugs dissolved in L-15 were introduced into the recording chamber with a peristaltic pump with a flow rate of 0.5–1.0 ml/min. In a few experiments, drugs were added by manual rapid bath replacement. In recordings where currents increased by 12% or more during perfusion of the drug the data were not selected for further analysis.

### Data Analysis

pClamp 8 and 10 (Molecular Devices) and Sigmaplot 11 (Systat Software, San Jose, CA, USA) were used to analyze voltage and current clamp data. MiniAnalysis software (v 6.0.3, Synaptosoft, Decatur, GA, USA) was used to analyze action potentials (magnitudes ≥30 mV, measured from the peak of an action potential to trough of the afterhyperpolarization potential). Statistical significance was determined using the Students’ *t* test (different populations), paired *t*-test (same population; before and after) and Wilcoxon Signed Rank test or Mann-Whitney Rank Sum Test (when data were not normally distributed). Values presented are means ± standard error of the mean (SEM) or medians. For all tests of statistically significant change, a result was deemed significant when *P* < 0.05 (denoted by*).

## Results

### Calyx K^+^ Currents Show Kinetic Differences Between Zones in the Utricle and Crista

**Figure 1:**
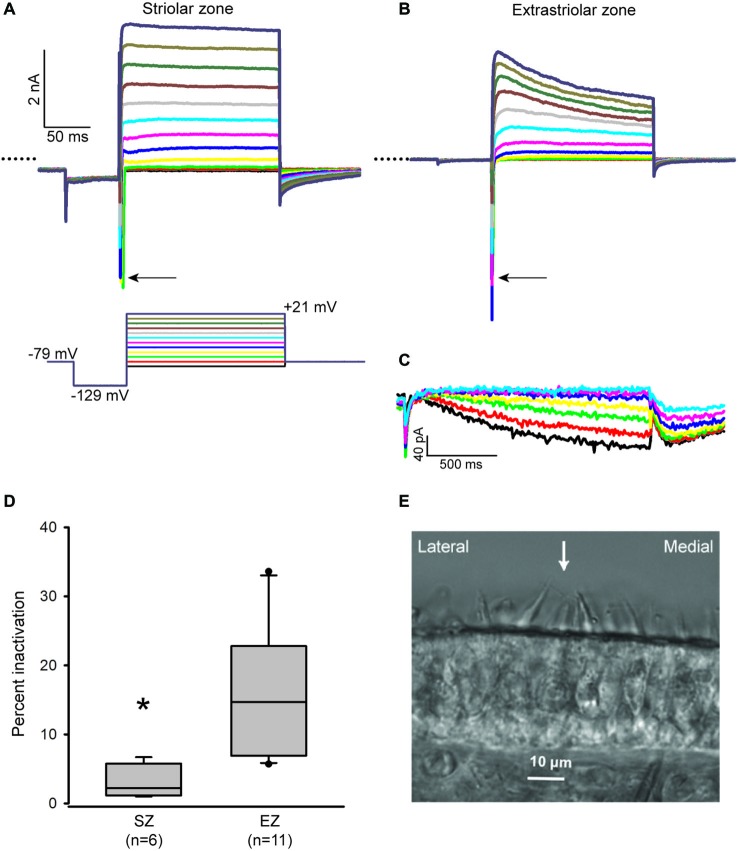
**Inactivation properties of outward K^+^ currents vary with zone in the utricle.** (**A** and **B**) Representative currents from a P17 striolar and P18 extrastriolar calyx (slices). Calyces were held at −79 mV, stepped to −129 mV and then stepped from −89 to +21 mV in 10 mV increments (voltage protocol shown below the striolar current traces). Typical fast inward Na^+^ currents (arrows) are present in both calyces. Dotted lines indicate zero current. Scale in **(B)** is the same as **(A)**. **(C)** HCN-mediated currents (I*_h_*) in response to incrementing 5 mV voltage steps (1.5 s duration) from −139 mV to −109 mV (same extrastriolar calyx as **B**). **(D)** Outward K^+^ current measured at the end of a 40 ms duration voltage step to +21 mV was compared to peak current to calculate inactivation. Box plots show % inactivation for striolar zone (SZ) and extrastriolar zone (EZ) calyces. Median inactivation was 14.7% in EZ calyces (*n* = 11), significantly greater than the value of 2.2% in SZ calyces (*n* = 6; *P* < 0.01, Mann-Whitney Rank Sum Test). **(E)** Differential interference contrast image of a transverse slice cut from the mid-region of a P20 gerbil utricle. Polarity reversal is indicated by the opposing hair bundles in focus with an arrow between them. The striola is defined as a band ~100 μM in width and situated medial to the LPR.

We recently developed a technique for making transverse slices through the gerbil crista, which allowed properties of calyces in central and PZ to be compared using whole cell patch clamp recordings (Meredith and Rennie, 2015). We have now applied this technique to the utricle, which is an otolith organ responsible for gravity sensing. Two distinct zones in the utricle are recognized; the striola is composed of a central band of hair cells and nerve terminals which is surrounded by the extrastriola (peripheral) region of cells. These two regions differ in several morphological features (Eatock and Songer, 2011). Calyx afferents within the striola are immunoreactive for the calcium-binding protein calretinin (Leonard and Kevetter, 2002; Desai et al., 2005b; Li et al., 2008) and have irregular firing characteristics (Goldberg, 2000). Hair bundles reverse their orientation around a line of polarity reversal (LPR) which defines the lateral border of the striola (Li et al., 2008). In the gerbil utricle, the striola is approximately 100 μM wide and more than 600 μM in length (Desai et al., 2005b). Hair bundle tall edges can be seen facing each other in Figure 1E on either side of the arrow. We made the assumption that the striola runs just medial to the LPR (Li et al., 2008) and classified calyces located within five hair cells medial to the LPR as belonging to the striola. Cells well outside of this zone and within 12 cells of the edge of the sensory epithelium were classified as “extrastriolar.”

Whole cell recordings were obtained from calyces innervating type I hair cells in utricular slices. Voltage steps revealed inward Na^+^ currents and outwardly rectifying K^+^ currents (Figures 1A,B) in addition to *I*_h_, a current activated at hyperpolarized membrane potentials and mediated by HCN channels (Figure 1C). In voltage clamp we found that outward K^+^ currents showed greater inactivation in extrastriolar zone (EZ) calyces compared to striolar zone (SZ) calyces (Figure 1D). Currents were measured at the end of a 40 ms duration voltage step (Figure 1D) to be consistent with previous measurements in crista calyces (Meredith and Rennie, 2015). We found that K^+^ currents in peripheral calyces show a greater degree of inactivation than central calyces in the crista (Meredith and Rennie, 2015) and confirmed a similar topographic heterogeneity with respect to K^+^ currents in the utricle.

### Effect of Dendrotoxins on Calyx Responses

**Figure 2:**
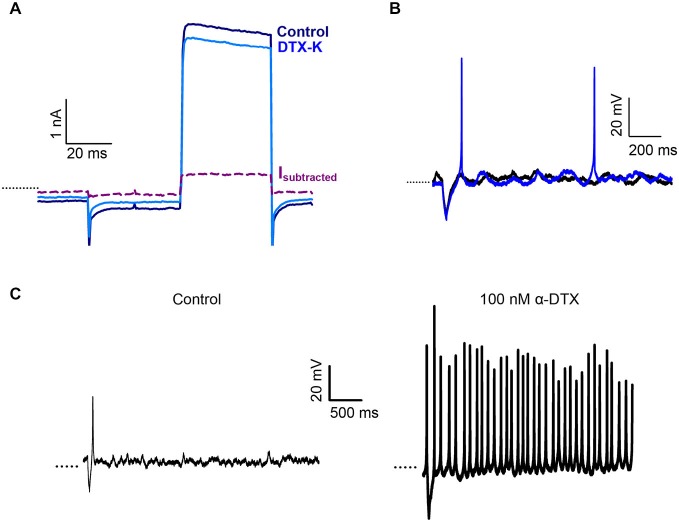
**Dendrotoxin-K (DTX-K) blocks a non-inactivating current in CZ calyces. (A)** 400 nM DTX-K blocked a portion of the outward K^+^ current in a CZ calyx (P25, slice). Current is in response to a +21 mV step (40 ms duration). The DTX-K-sensitive current (I*_subtracted_*) activated rapidly and showed no inactivation. **(B)** DTX-K (400 nM) increased the number of action potentials in a CZ calyx (P28, slice) from a control value of 0 (black) to 2 (blue). **(C)** Exposure to 100 nM α-DTX resulted in a change from one action potential evoked after a short, hyperpolarizing current injection (control, left) to tonic firing in a PZ calyx (P22, slice). Dotted line indicates −60 mV.

We previously showed that the snake toxin α-dendrotoxin (α-DTX) blocked a K^+^ current in centrally located calyces of the gerbil crista, but had no significant effect in peripheral calyces (Meredith and Rennie, 2015). α-DTX is a known blocker of the subunits Kv1.1, 1.2 and Kv1.6 and the biochemical presence of these three subunits has been reported in the vestibular ganglion and periphery (Lysakowski et al., 2011; Iwasaki et al., 2012). We used additional selective blockers of Kv1 channel subunits to probe K^+^ ion channels in the afferent calyx terminal. The effect of dendrotoxin-K (DTX-K), a selective blocker of Kv1.1 and Kv1.2 channels, was tested on crista calyx terminals (Figure 2). DTX-K (400 nM) reduced whole cell outward current in calyces from central regions by a mean value of 10.6 ± 1.9% (*n* = 6). The current blocked by DTX-K showed no inactivation over the duration of the pulse (Figure 2A), as reported previously for α-dendrotoxin-sensitive current in CZ calyces. PZ calyces did not show an appreciable change in outward current in response to DTX-K (current decreased on average by 1.0 ± 3.0%, *n* = 4).

DTX-K blocked current in crista CZ calyces, but not PZ calyces consistent with our previous observations with α-DTX (Meredith and Rennie, 2015). In current clamp DTX-K increased the number of action potentials evoked following a stimulus as shown in Figure 2B. Firing in the presence of α-DTX or DTX-K increased from a median of 1 Hz to 2 Hz in CZ calyces (*P* < 0.05, *n* = 6, Wilcoxon Signed Rank test). Interestingly, although neither of the dendrotoxins blocked outward current in PZ calyces, an increase in firing was also seen for PZ calyces in response to α-DTX (Figure 2C). These results suggest that Kv1.1 and/or Kv1.2 channels contribute to a resting conductance which predominates in CZ calyces and that acts to reduce excitability in these afferents. PZ calyx afferents are dimorphic terminals that can branch extensively (Eatock and Songer, 2011). Since an increase in firing was seen in PZ calyces, but no effect was seen in voltage clamp, the dendrotoxin effect may occur at some distance from the recording site.

### The Kv1 Channel Blockers Margatoxin and CP-339, 818

**Figure 3:**
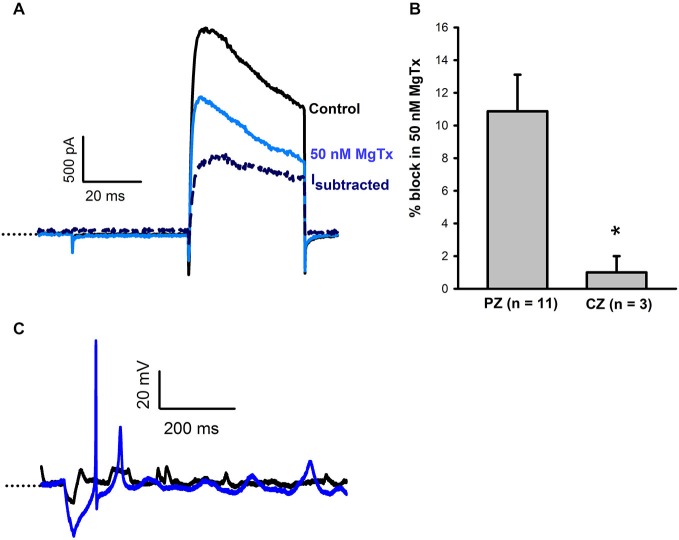
**Margatoxin (50 nM) blocks a slowly activating, partially inactivating current. (A)** In a PZ calyx (P27, dissociated), 50 nM margatoxin blocked current elicited in response to a +21 mV voltage step. The margatoxin-sensitive current (I*_subtracted_*) showed partial inactivation during the 40 ms pulse (navy, dashed line). **(B)** Margatoxin blocked current in PZ calyces (*n* = 11), but not CZ calyces (*n* = 3, *P* < 0.05, *t*-test). **(C)** Under control conditions, an action potential was not evoked in a PZ calyx (black trace, P23, cut end). Following exposure to 50 nM margatoxin an action potential followed by a second spike of smaller amplitude were evoked. Dotted line indicates −60 mV.

Margatoxin is reported to selectively block Kv1.3 and Kv1.6 channels (Garcia-Calvo et al., 1993) and was previously shown to block a component of the voltage-dependent outward current in vestibular ganglion cells (VGCs; Chabbert et al., 2001; Iwasaki et al., 2008). Margatoxin (50 nM) was applied to calyces from CZ and PZ of the crista (Figure 3). In 11 PZ calyces margatoxin produced a mean block of 10.9 ± 2.2% of outward current (Figure 3B). This differed significantly from CZ cells which showed little response to margatoxin (Figure 3B). The margatoxin-sensitive current in PZ calyces was different from the dendrotoxin-sensitive current, having slower activation kinetics and showing partial inactivation during the test pulse (Figure 3A). Therefore margatoxin-sensitive K^+^ currents may contribute to the greater degree of inactivation in PZ cells. In one PZ calyx tested in current clamp, firing increased in the presence of margatoxin (Figure 3C).

**Figure 4:**
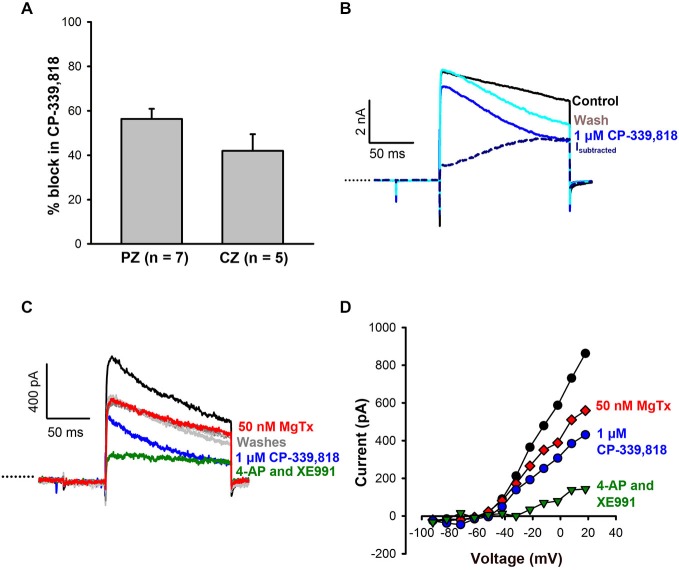
**CP-339, 818 blocks a non-inactivating component of the current in central and peripheral zone calyces. (A)** No significant difference in mean reduction in current (measured at the end of the +21 mV step) was seen in CZ and PZ calyces. **(B)** Currents in response to a +21 mV voltage step for control (black) and after application of 1 μM CP-339, 818 (blue) in a crista CZ calyx (slice). The CP-339, 818-sensitive current (I*_subtracted_*) showed an instantaneous component followed by a slow activation and no inactivation throughout the voltage step. **(C)** The CP-339, 818-sensitive current is distinct from the margatoxin-sensitive current. Control current (black trace) in an isolated crista calyx in response to a voltage step to +19 mV. Application of 50 nM margatoxin reduced the outward current (red). No recovery was seen after return to control solution (dashed gray line). Application of 1 μM CP-339, 818 produced a larger reduction in current and recovery was seen during a wash (light gray trace). A subsequent application of 100 μM 4-AP and 20 μM XE991, which block Kv1 and KCNQ channels respectively, greatly reduced outward currents (green trace), followed by recovery in control solution (dark gray trace). **(D)** Current-voltage *(I-V)* plot for cell in **(C)** showing peak currents following application of margatoxin, CP-339, 818 and a combination of 100 μM 4-AP and 20 μM XE991.

To investigate possible contributions from other Kv1 subunits, CP-339, 818, a nonpeptide blocker of Kv1.3 and Kv1.4 channels (Nguyen et al., 1996), was tested on calyces. CP-339, 818 blocked a substantial portion of the outward current in both CZ (42 ± 7.5% of current) and PZ calyces (56 ± 4.5% of current) at concentrations ranging from 1–4 μM (Figure 4A). Current subtractions revealed CP-339, 818 blocked a slowly activating current which did not inactivate (Figure 4B). CP-339, 818 also reduced the Na^+^ current, so we could not study the effect of this drug on firing. The current blocked by CP-339, 818 was distinct from the current blocked by margatoxin, as shown in Figures 4C,D. In this calyx margatoxin irreversibly reduced the outward current. Subsequent application of 1 μM CP-339, 818 produced a further reversible reduction in outward current, suggesting involvement of different K^+^ channel subunits. A cocktail of 100 μM 4-AP and 20 μM XE991, blocking both Kv1 and KCNQ channels, produced the greatest reduction in outward current.

## Discussion

### Signal Coding by Vestibular Afferents

The peripheral vestibular end organs comprise the utricle, saccule and the cristae of the semicircular canals. Initial processing of vestibular signals occurs when type I and type II hair cells convert mechanical signals into electrical receptor potentials which modulate release of glutamate onto afferent terminals. Vestibular afferent terminals exhibit three distinct dendritic morphologies. Calyx-only dendrites innervate one or more type I hair cells, bouton dendrites innervate only type II hair cells and dimorphic afferent dendrites contact both hair cell types. Calyx-only terminals are found in CZ, bouton terminals populate PZ and dimorphic fibers are found throughout the sensory epithelium. Afferent properties differ between central and peripheral areas of the crista and striolar and extrastriolar regions of the utricle (Goldberg, 2000; Eatock and Songer, 2011). Afferents in central locations of vestibular epithelia typically have irregular spontaneous discharge and phasic response dynamics, whereas peripheral afferents have more regular firing patterns and tonic response properties (Goldberg, 2000; Eatock and Songer, 2011). Corresponding phasic and tonic firing patterns are also evident in VGCs; however *in vitro* preparations of VGCs show evoked and not spontaneous firing of action potentials (Iwasaki et al., 2008; Pérez et al., 2009; Kalluri et al., 2010). The coding strategies used by afferent nerves in the vestibular system and how they change with altered input, such as changing gravitational signals, are not well understood. However, emerging data suggest that ion channels at or close to the spike initiation zone in vestibular afferent terminals are key contributors to action potential generation (Lysakowski et al., 2011). Data presented here show differences in K^+^ current kinetics and composition between different zones of the crista and utricle that may impact the types of signals carried by afferents. Central calyx units have the most irregular firing patterns and low-voltage activated K^+^ channels may contribute to irregularity and phasic responses. Dendrotoxin-sensitive currents that show little inactivation may enable detection of high-frequency signals and be important for generating rapid vestibular-driven reflexes. The greater inactivation of K^+^ currents in peripheral and extrastriolar calyces could contribute to the tonic activity of regular afferents, which may be better suited to signaling long duration stimuli.

### K^+^ Currents in Calyces

Recent work suggests conductances intrinsic to calyces contribute to spontaneous firing patterns in vestibular afferents (Meredith et al., 2012; Horwitz et al., 2014; Meredith and Rennie, 2015). In the crista, outward K^+^ currents showed greater inactivation in peripheral calyces compared to central calyces (Meredith and Rennie, 2015) and as reported here we also find significant zonal differences in inactivation in the utricle. Median K^+^ current inactivation in extrastriolar calyces was 15%, significantly greater than median inactivation observed in striolar calyces (2%). Further experiments are needed to determine if K^+^ current inactivation properties and magnitudes show further gradients across vestibular epithelia. Our results differ from a recent study in early postnatal mouse utricle, where no clear regional differences in K^+^ or Na^+^ currents in calyx-bearing afferents were seen (Horwitz et al., 2014).

Outward K^+^ currents in isolated calyces were previously shown to have underlying components sensitive to the K^+^ channel blockers tetraethylammonium (TEA) and 4-aminopyridine (4-AP, 0.25–1.25 mM; Dhawan et al., 2010). TEA blocked a slowly activating, slowly inactivating current, whereas 4-AP blocked a more rapidly activating and inactivating component of the current (Dhawan et al., 2010). The TEA-sensitive current is likely mediated at least in part by KCNQ channels (Hurley et al., 2006; Rennie and Streeter, 2006; Meredith and Rennie, 2015), whereas 4-AP is a known broad spectrum blocker of Kv1 channels (Coetzee et al., 1999). K^+^ currents in isolated calyces showed considerable variation in their inactivation kinetics, suggesting varying contributions from different subunits, but the original epithelial position of cells was unknown (Dhawan et al., 2010). We subsequently showed a differential sensitivity to 4-AP in calyces of the crista, where 4-AP at low concentrations (10–100 μM) blocked more current in peripherally located calyces compared to central calyces, supporting a differential distribution of Kv1 channels. At higher concentrations 4-AP blocked a greater portion of the total current in both zones of the crista and no significant difference between zones was seen (Meredith and Rennie, 2015).

Results reported here with the more selective blockers DTX-K and margatoxin reveal further regional variations in Kv1 channel subunits in vestibular epithelia. In voltage clamp DTX-K, a selective blocker of Kv1.1 and Kv1.2 subunits, inhibited a rapidly activating, non-inactivating current in CZ crista calyces, but not PZ calyces. However we found that the dendrotoxins increased the frequency of firing when applied to both CZ and PZ calyces. In VGCs, α-DTX reduced outward K^+^ current and increased firing in phasic but not tonic VGCs (Iwasaki et al., 2008). The same authors observed a third class of VGCs with intermediate firing properties that responded to application of α-DTX, DTX-K or margatoxin with an increase in firing frequency. Phasic VGCs may correspond to irregularly firing calyx afferents and tonic VGCs to regularly firing afferents. Taken together the results support a role for Kv1.1 and/or Kv1.2 channels in reducing excitability and altering response dynamics in central calyx-bearing afferents with irregular firing patterns. Further studies within the central zone are required to selectively investigate “pure” calyx afferents, contacting only type I hair cells and dimorphic fibers, which receive input from both calyces and collateral dendrites terminating as boutons on type II hair cells.

We found no evidence for dendrotoxin-sensitive currents in PZ calyces, whose membrane properties were dominated by inactivating K^+^ currents. Interestingly an increase in firing was seen in PZ calyces in response to dendrotoxin application. We speculate that dendrotoxin may block channels in these dimorphic afferents at a location distal to the calyx, such as the bouton afferent.

Margatoxin, a known blocker of Kv1.3 and Kv1.6 channels at nanomolar concentrations, caused a greater reduction of current in calyces that expressed K^+^ currents with greater inactivation. CP-339, 818, which is reported to block Kv1.3 and Kv1.4 at high nanomolar concentrations (Nguyen et al., 1996), reduced current in both CZ and PZ calyces. We used CP-339, 818 at concentrations that should not block other K^+^ channels belonging to the Kv1, Kv3, and Kv4 families (Nguyen et al., 1996). The CP-339, 818-sensitive current was kinetically different from the current blocked by margatoxin. Given the differences in block, margatoxin may inhibit Kv1.6-mediated currents, whereas CP-339, 818 may block Kv1.4 channels. Interestingly a current sensitive to 1 μM CP-339, 818 was recently described in spiral ganglion neurons (SGN) and was suggested to be mediated by Kv1.4 channels (Wang et al., 2013).

### The Role of Kv1 Channels in Firing in Vestibular and Auditory Afferents

Our goal is to elucidate the roles of different K^+^ channels in spike generation and firing in vestibular afferent calyces. In other neurons, Kv1 channels mediate delayed rectifier currents that typically open with small depolarizations at or below the cell resting membrane potential. Efflux of K^+^ through Kv1 channels hyperpolarizes the cell membrane and limits excitability. A recent study in rat crista demonstrated immunolabelling for Kv1.1 and Kv1.2 channels on the inner face of calyces and at juxtaparaheminodal locations, near the first internode of the vestibular afferent neuron (Lysakowski et al., 2011). Kv1.2 immunostaining was also reported in calyces of human and mouse vestibular epithelia (Hotchkiss et al., 2005). Kv1.1, Kv1.2, and Kv1.6 were present in rat VGCs, but a decrease in Kv1.6 biochemical expression after the first postnatal week prompted speculation that changes in Kv1.6 expression may correlate with alterations in afferent firing patterns during development (Iwasaki et al., 2012). α-DTX, margatoxin and 4-AP also blocked components of the whole cell current in VGCs (Chabbert et al., 2001; Iwasaki et al., 2008). In current clamp, evoked firing of action potentials in VGCs converted from phasic to tonic in response to α-DTX (Iwasaki et al., 2008; Kalluri et al., 2010).

In the auditory system, SGN carry sound information from inner hair cells to the brain and show substantial variations in their electrophysiological properties (Taberner and Liberman, 2005). Kv1 channels are important determinants of firing and recent data suggest Kv1 and HCN channels contribute to heterogeneity in SGN action potential threshold and membrane potential (Wang et al., 2013; Liu et al., 2014). An increased expression of Kv1.1 and Kv1.2 channels was seen in basal neurons and α-DTX application converted action potential firing from rapid to slow accommodation (Liu et al., 2014). Therefore Kv1 channels appear to be important for setting the membrane potential at which inner ear afferents generate action potentials and determining whether a neuron fires single or multiple action potentials.

### Spaceflight-induced Changes in the Vestibular System

The vestibular system is important for many aspects of brain function including reflexes, spatial perception and motor coordination. In SAS, manual performance and cognitive tasks can be greatly impaired and episodes of severe motion sickness can result (Lackner, 2014). Pharmacological agents to combat motion sickness exist, but are not particularly selective and can have unwanted side-effects such as drowsiness (Lackner, 2014). Motion sickness does not occur without an intact vestibular system (Yates et al., 1998). Spaceflight may drive changes in the activity of ion channels in the otolith organs and cristae during adaptation to the new environment that may impact motion sickness. Ground-based studies have shown that exposure to hypergravity altered the developmental time course of hair cell Na^+^ currents (Brugeaud et al., 2006) and resulted in an increase in K^+^ current magnitude in early postnatal type I and type II hair cells of the rat utricle, although underlying K^+^ channel subtypes were not identified (Chabbert et al., 2003). In frog semicircular canal, short periods of microgravity resulted in decreased magnitudes of both calcium and delayed rectifier potassium current in type II hair cells (Martini et al., 2009). Changes in the gain of primary vestibular afferents (Correia et al., 1992) and changes in the number of ribbon synapses per hair cell have also been reported following spaceflight (Ross, 1994, 2000). These studies indicate that changes in the gravitational field can induce alterations in hair cell conductances and likely changes in synaptic strength (plasticity) in the vestibular periphery. Although hypergravity-induced changes in hair cell conductances during development were seen (Chabbert et al., 2003; Brugeaud et al., 2006), investigations in adult mammalian models are lacking.

### 4-AP and Labyrinthectomy

Since 4-AP blocks a variety of potassium channels, it has been used in the treatment of neurological conditions including cerebellar disorders, ataxias and central ocular motor disorders (Strupp et al., 2011). In episodic ataxia type 1, mutations in the KCNA1 gene occur resulting in defective Kv1.1 channels, increased neuronal excitability, prolongation of action potentials and in some cases deafness (Jen, 2008; Tomlinson et al., 2013). These channels are widely expressed in the central nervous system and as shown here may also be present in vestibular afferent terminals. In episodic ataxia type 2 (EA2), 4-AP restores pacemaking activity in Purkinje cells, likely through an effect on Kv1 channels (Alviña and Khodakhah, 2010). 4-AP has also been used in patients with EA2 with promising results (Strupp et al., 2011).

The adult vestibular system must retain plasticity in order for recalibration to changing signals to occur. At the medial vestibular nucleus, plasticity changes are reported in response to removal of vestibular afferents (Smith and Curthoys, 1989). In chick tangential nucleus, changes in dendrotoxin-sensitive currents were observed following unilateral vestibular ganglionectomy (Shao et al., 2009). In rats a decrease in spike discharge was seen on the side of the lesion, whereas an increase in spike rate occurred on the intact side following labyrinthectomy (Smith and Curthoys, 1989). Mechanisms of these adaptive changes are unresolved, but may involve changes in K^+^ channel subunit composition. When rats underwent unilateral labyrinthectomy, daily administration of 4-AP resulted in an early significant improvement in postural imbalance at 1–3 days post-surgery compared to controls; the effect presumably involved K^+^ channels in vestibular neurons (Beck et al., 2014). Changes in Kv1 channel expression could be involved and more selective Kv1 channel blockers may be useful in therapeutic treatment of disorders and recalibration of the vestibular system.

## Conflict of Interest Statement

The authors declare that the research was conducted in the absence of any commercial or financial relationships that could be construed as a potential conflict of interest.

## References

[B1] Alviña K. Khodakhah K. (2010). The therapeutic mode of action of 4-aminopyridine in cerebellar ataxia. J. Neurosci. 30, 7258–7268. 10.1523/JNEUROSCI.3582-09.2010 20505092 PMC2909847

[B2] Beck R. Günther L. Xiong G. Potschka H. Böning G. Bartenstein P.. (2014). The mixed blessing of treating symptoms in acute vestibular failure-evidence from a 4-aminopyridine experiment. Exp. Neurol. 261, 638–645. 10.1016/j.expneurol.2014.08.013 25157903

[B3] Brugeaud A. Gaboyard-Niay S. Puel J. L. Chabbert C. (2006). Hypergravity affects the developmental expression of voltage-gated sodium current in utricular hair cells. Neuroreport 17, 1697–1701. 10.1097/01.wnr.0000239961.98813.19 17047456

[B4] Chabbert C. Brugeaud A. Lennan G. Lehouelleur J. Sans A. (2003). Electrophysiological properties of the utricular primary transducer are modified during development under hypergravity. Eur. J. Neurosci. 17, 2497–2500. 10.1046/j.1460-9568.2003.02682.x 12814383

[B5] Chabbert C. Chambard J. M. Sans A. Desmadryl G. (2001). Three types of depolarization-activated potassium currents in acutely isolated mouse vestibular neurons. J. Neurophysiol. 85, 1017–1026. 11247971 10.1152/jn.2001.85.3.1017

[B6] Coetzee W. A. Amarillo Y. Chiu J. Chow A. Lau D. Mccormack T. (1999). Molecular diversity of K^+^ channels. Ann. N Y Acad. Sci. 868, 233–285. 10.1111/j.1749-6632.1999.tb11293.x 10414301

[B7] Correia M. J. Perachio A. A. Dickman J. D. Kozlovskaya I. B. Sirota M. G. Yakushin S. B.. (1992). Changes in monkey horizontal semicircular canal afferent responses after spaceflight. J. Appl. Physiol. (1985) 73, 112S–120S. 1326513 10.1152/jappl.1992.73.2.S112

[B8] Desai S. S. Ali H. Lysakowski A. (2005a). Comparative morphology of rodent vestibular periphery. II. Cristae ampullares. J. Neurophysiol. 93, 267–280. 10.1152/jn.00747.2003 15240768 PMC12513555

[B9] Desai S. S. Zeh C. Lysakowski A. (2005b). Comparative morphology of rodent vestibular periphery. I. Saccular and utricular maculae. J. Neurophysiol. 93, 251–266. 10.1152/jn.00746.2003 15240767 PMC12456082

[B10] Dhawan R. Mann S. E. Meredith F. L. Rennie K. J. (2010). K^+^ currents in isolated vestibular afferent calyx terminals. J. Assoc. Res. Otolaryngol. 11, 463–476. 10.1007/s10162-010-0213-8 20407915 PMC2914245

[B11] Eatock R. A. Songer J. E. (2011). Vestibular hair cells and afferents: two channels for head motion signals. Annu. Rev. Neurosci. 34, 501–534. 10.1146/annurev-neuro-061010-113710 21469959

[B12] Garcia-Calvo M. Leonard R. J. Novick J. Stevens S. P. Schmalhofer W. Kaczorowski G. J.. (1993). Purification, characterization and biosynthesis of margatoxin, a component of Centruroides margaritatus venom that selectively inhibits voltage-dependent potassium channels. J. Biol. Chem. 268, 18866–18874. 8360176

[B13] Goldberg J. M. (2000). Afferent diversity and the organization of central vestibular pathways. Exp. Brain Res. 130, 277–297. 10.1007/s002210050033 10706428 PMC3731078

[B14] Horwitz G. C. Risner-Janiczek J. R. Holt J. R. (2014). Mechanotransduction and hyperpolarization-activated currents contribute to spontaneous activity in mouse vestibular ganglion neurons. J. Gen. Physiol. 143, 481–497. 10.1085/jgp.201311126 24638995 PMC3971655

[B15] Hotchkiss K. Harvey M. Pacheco M. Sokolowski B. (2005). Ion channel proteins in mouse and human vestibular tissue. Otolaryngol. Head Neck Surg. 132, 916–923. 10.1016/j.otohns.2005.01.022 15944564

[B16] Hurley K. M. Gaboyard S. Zhong M. Price S. D. Wooltorton J. R. Lysakowski A.. (2006). M-like K^+^ currents in type I hair cells and calyx afferent endings of the developing rat utricle. J. Neurosci. 26, 10253–10269. 10.1523/jneurosci.2596-06.2006 17021181 PMC6674627

[B17] Iwasaki S. Chihara Y. Komuta Y. Ito K. Sahara Y. (2008). Low-voltage-activated potassium channels underlie the regulation of intrinsic firing properties of rat vestibular ganglion cells. J. Neurophysiol. 100, 2192–2204. 10.1152/jn.01240.2007 18632889

[B18] Iwasaki S. Nakajima T. Chihara Y. Inoue A. Fujimoto C. Yamasoba T. (2012). Developmental changes in the expression of Kv1 potassium channels in rat vestibular ganglion cells. Brain Res. 1429, 29–35. 10.1016/j.brainres.2011.10.015 22079321

[B19] Jan L. Y. Jan Y. N. (2012). Voltage-gated potassium channels and the diversity of electrical signalling. J. Physiol. 590, 2591–2599. 10.1113/jphysiol.2011.224212 22431339 PMC3424718

[B20] Jen J. C. (2008). Recent advances in the genetics of recurrent vertigo and vestibulopathy. Curr. Opin. Neurol. 21, 3–7. 10.1097/WCO.0b013e3282f41ca0 18180645

[B21] Kalluri R. Xue J. Eatock R. A. (2010). Ion channels set spike timing regularity of mammalian vestibular afferent neurons. J. Neurophysiol. 104, 2034–2051. 10.1152/jn.00396.2010 20660422 PMC2957450

[B22] Lackner J. R. (2014). Motion sickness: more than nausea and vomiting. Exp. Brain Res. 232, 2493–2510. 10.1007/s00221-014-4008-8 24961738 PMC4112051

[B23] Leonard R. B. Kevetter G. A. (2002). Molecular probes of the vestibular nerve. I. Peripheral termination patterns of calretinin, calbindin and peripherin containing fibers. Brain Res. 928, 8–17. 10.1016/s0006-8993(01)03268-1 11844467

[B24] Li A. Xue J. Peterson E. H. (2008). Architecture of the mouse utricle: macular organization and hair bundle heights. J. Neurophysiol. 99, 718–733. 10.1152/jn.00831.2007 18046005

[B25] Lindeman H. H. (1969). Studies on the morphology of the sensory regions of the vestibular apparatus with 45 figures. Ergeb. Anat. Entwicklungsgesch. 42, 1–113. 5310109

[B26] Liu Q. Lee E. Davis R. L. (2014). Heterogeneous intrinsic excitability of murine spiral ganglion neurons is determined by Kv1 and HCN channels. Neuroscience 257, 96–110. 10.1016/j.neuroscience.2013.10.065 24200924 PMC3897933

[B28] Lysakowski A. Gaboyard-Niay S. Calin-Jageman I. Chatlani S. Price S. D. Eatock R. A. (2011). Molecular microdomains in a sensory terminal, the vestibular calyx ending. J. Neurosci. 31, 10101–10114. 10.1523/JNEUROSCI.0521-11.2011 21734302 PMC3276652

[B27] Lysakowski A. Goldberg J. M. (2008). Ultrastructural analysis of the cristae ampullares in the squirrel monkey (Saimiri sciureus). J. Comp. Neurol. 511, 47–64. 10.1002/cne.21827 18729176 PMC2828494

[B29] Martini M. Canella R. Leparulo A. Prigioni I. Fesce R. Rossi M. L. (2009). Ionic currents in hair cells dissociated from frog semicircular canals after preconditioning under microgravity conditions. Am. J. Physiol. Regul. Integr. Comp. Physiol. 296, R1585–R1597. 10.1152/ajpregu.90981.2008 19244579

[B31] Meredith F. L. Benke T. A. Rennie K. J. (2012). Hyperpolarization-activated current (I(h)) in vestibular calyx terminals: characterization and role in shaping postsynaptic events. J. Assoc. Res. Otolaryngol. 13, 745–758. 10.1007/s10162-012-0342-3 22825486 PMC3505587

[B32] Meredith F. L. Li G. Q. Rennie K. J. (2011). Postnatal expression of an apamin-sensitive k(ca) current in vestibular calyx terminals. J. Membr. Biol. 244, 81–91. 10.1007/s00232-011-9400-8 22057903 PMC3242503

[B30] Meredith F. L. Rennie K. J. (2015). Zonal variations in K^+^ currents in vestibular crista calyx terminals. J. Neurophysiol. 113, 264–276. 10.1152/jn.00399.2014 25343781 PMC5005277

[B33] Nguyen A. Kath J. C. Hanson D. C. Biggers M. S. Canniff P. C. Donovan C. B.. (1996). Novel nonpeptide agents potently block the C-type inactivated conformation of Kv1.3 and suppress T cell activation. Mol. Pharmacol. 50,1672–1679. 8967992

[B34] Pérez C. Limón A. Vega R. Soto E. (2009). The muscarinic inhibition of the potassium M-current modulates the action-potential discharge in the vestibular primary-afferent neurons of the rat. Neuroscience 158, 1662–1674. 10.1016/j.neuroscience.2008.11.023 19095045

[B35] Rennie K. J. Streeter M. A. (2006). Voltage-dependent currents in isolated vestibular afferent calyx terminals. J. Neurophysiol. 95, 26–32. 10.1152/jn.00641.2005 16162827

[B36] Rizzo-Sierra C. V. Leon-Sarmiento F. E. (2011). Pathophysiology of movement disorders due to gravity transitions: the channelopathy linkage in human balance and locomotion. Med. Hypotheses 77, 97–100. 10.1016/j.mehy.2011.03.036 21482451

[B37] Ross M. D. (1994). A spaceflight study of synaptic plasticity in adult rat vestibular maculas. Acta Otolaryngol. Suppl. 516, 1–14. 7976320

[B38] Ross M. D. (2000). Changes in ribbon synapses and rough endoplasmic reticulum of rat utricular macular hair cells in weightlessness. Acta Otolaryngol. 120, 490–499. 10.1080/000164800750045983 10958400

[B39] Shao M. Popratiloff A. Hirsch J. C. Peusner K. D. (2009). Presynaptic and postsynaptic ion channel expression in vestibular nuclei neurons after unilateral vestibular deafferentation. J. Vestib. Res. 19, 191–200. 10.3233/VES-2009-0348 20495236

[B40] Smith P. F. Curthoys I. S. (1989). Mechanisms of recovery following unilateral labyrinthectomy: a review. Brain Res. Brain Res. Rev. 14, 155–180. 10.1016/0165-0173(89)90013-1 2665890

[B41] Soto E. Vega R. (2010). Neuropharmacology of vestibular system disorders. Curr. Neuropharmacol. 8, 26–40. 10.2174/157015910790909511 20808544 PMC2866460

[B42] Strupp M. Kalla R. Claassen J. Adrion C. Mansmann U. Klopstock T.. (2011). A randomized trial of 4-aminopyridine in EA2 and related familial episodic ataxias. Neurology 77, 269–275. 10.1212/WNL.0b013e318225ab07 21734179 PMC3136055

[B43] Taberner A. M. Liberman M. C. (2005). Response properties of single auditory nerve fibers in the mouse. J. Neurophysiol. 93, 557–569. 10.1152/jn.00574.2004 15456804

[B44] Tomlinson S. E. Rajakulendran S. Tan S. V. Graves T. D. Bamiou D. E. Labrum R. W.. (2013). Clinical, genetic, neurophysiological and functional study of new mutations in episodic ataxia type 1. J. Neurol. Neurosurg. Psychiatry 84, 1107–1112. 10.1136/jnnp-2012-304131 23349320 PMC4332158

[B45] Wang W. Kim H. J. Lv P. Tempel B. Yamoah E. N. (2013). Association of the Kv1 family of K^+^ channels and their functional blueprint in the properties of auditory neurons as revealed by genetic and functional analyses. J. Neurophysiol. 110, 1751–1764. 10.1152/jn.00290.2013 23864368 PMC3798938

[B46] Yates B. J. Miller A. D. Lucot J. B. (1998). Physiological basis and pharmacology of motion sickness: an update. Brain Res. Bull. 47, 395–406. 10.1016/s0361-9230(98)00092-6 10052567

[B47] Zdebik A. A. Wangemann P. Jentsch T. J. (2009). Potassium ion movement in the inner ear: insights from genetic disease and mouse models. Physiology (Bethesda) 24, 307–316. 10.1152/physiol.00018.2009 19815857 PMC4415853

